# Differences in Electronic Personal Health Information Tool Use Between Rural and Urban Cancer Patients in the United States: Secondary Data Analysis

**DOI:** 10.2196/17352

**Published:** 2020-08-10

**Authors:** Alexandra Greenberg-Worisek, Liaa Ferede, Joyce Balls-Berry, Ian Marigi, Emily Valentin Mendez, Numra Bajwa, Melody Ouk, Minerva Orellana, Felicity Enders

**Affiliations:** 1 Mayo Clinic Rochester, MN United States; 2 University of Minnesota Rochester Rochester, MN United States; 3 Department of Neurologic Surgery Mayo Clinic Rochester, MN United States; 4 Washington University in St Louis St Louis, MO United States; 5 University of Puerto Rico School of Medicine San Juan, Puerto Rico United States Minor Outlying Islands

**Keywords:** cancer, patient engagement, health research, digital divide, disparities

## Abstract

**Background:**

Studies have previously shown that rural cancer patients are diagnosed at later stages of disease. This delay is felt throughout treatment and follow-up, reflected in the fact that rural patients often have poorer clinical outcomes compared with their urban counterparts.

**Objective:**

Few studies have explored whether there is a difference in cancer patients’ current use of health information technology tools by residential location.

**Methods:**

Data from 7 cycles of the Health Information National Trends Survey (HINTS, 2003-2017) were merged and analyzed to examine whether differences exist in managing electronic personal health information (ePHI) and emailing health care providers among rural and urban cancer patients. Geographic location was categorized using Rural-Urban Continuum Codes (RUCCs). Bivariate analyses and multivariable logistic regression were used to determine whether associations existed between rural/urban residency and use of health information technology among cancer patients.

**Results:**

Of the 3031 cancer patients/survivors who responded across the 7 cycles of HINTS, 797 (26.9%) resided in rural areas. No difference was found between rural and urban cancer patients in having managed ePHI in the past 12 months (OR 0.78, 95% CI 0.43-1.40). Rural cancer patients were significantly less likely to email health care providers than their urban counterparts (OR 0.52, 95% CI 0.32-0.84).

**Conclusions:**

The digital divide between rural and urban cancer residents does not extend to general ePHI management; however, electronic communication with providers is significantly lower among rural cancer patients than urban cancer patients. Further research is needed to determine whether such disparities extend to other health information technology tools that might benefit rural cancer patients as well as other chronic conditions.

## Introduction

Patients with chronic diseases require complex and ongoing care. Specifically, patients with cancer diagnoses require frequent and deep contact with the health care system. This is a particular challenge for rural cancer patients, who must travel on average 48 miles to see their nearest health care provider in person [1,2]. The impact of distance from providers is felt throughout the cancer care continuum, from detection and diagnosis to treatment and follow-up care. For example, rural cancer patients have a significantly lower chance of receiving appropriate chemotherapy than their urban counterparts, due in part to distance and travel time [1,2]. Although many telemedicine centers were established to increase geographic access for rural patients, many are still too far for certain geographic populations [2,3].

The lack of access due to travel distance results in rural cancer patients participating less frequently in regular cancer screening than urban cancer patients, including screenings for more prevalent malignancies, such as breast, colon, and prostate cancer [1]. Due to the lower rates of patients in rural regions getting cancer screenings, they are more likely to be diagnosed with cancer at a later stage than patients who live in an urban region [1]. This may, in part, help to explain why cancer patients in rural regions have a higher mortality rate than cancer patients in urban regions [4]. Efforts have been made in recent years to use technology to creatively reach specific groups of patients in rural areas, such as telemedicine programs aimed at reaching rural Native American communities, or for certain specialties, including ambulatory, inpatient, and perinatal care [5-7].

Accompanying the rise of telemedicine has been increasing internet adoption nationwide, with studies reporting that access to the internet increased for all sociodemographic groups between 2003 and 2014 [8]. This is due in part to advances in technology, which allow individuals to access the internet more freely and on-demand using handheld and portable devices [9]. In parallel with these hardware and internet connectivity advances has been increasing adoption of electronic health records (EHRs) and electronic personal health information (ePHI) tools by health care providers; this has the potential to facilitate increased patient engagement and communication with health care providers [10]. Despite efforts to increase access to the internet and facilitate opportunities for remote interaction with the health care system, populations still lack internet access and connection quality, which affects their ability to access and use ePHI tools; this, in turn, may be further exacerbating the existing health information technology–related digital divide among rural and urban patients.

In this study, we sought to (1) determine the overall use of ePHI tools among cancer patients in urban and rural regions and (2) assess the rate of email communication between cancer patients in urban and rural regions and their health care provider. We hypothesized that urban cancer patients access their ePHI more frequently than rural cancer patients and urban cancer patients communicate via email with their health care provider more frequently than cancer patients in rural regions. To study the rural-urban disparity longitudinally and determine whether it was growing, we used multiple administrations of the National Cancer Institute’s Health Information National Trends Survey (HINTS) data.

## Methods

### Survey Population and Data Collection

HINTS is a nationally representative survey of noninstitutionalized adults over the age of 18 years in the United States. The survey includes a variety of health-related topics, such as the use of health technology and communication with health care providers. The mode of survey delivery varied across HINTS fieldings and included random digital dialing (RDD) and regular mail distribution. Data from years 2003, 2005, 2008, 2011, 2013, and 2017 were included in the survey. Surveys were distributed though RDD in 2003, 2005, and 2008. Physical mail distribution occurred in years 2008 (in parallel with RDD), 2011, 2013, and 2017. The response rate of random digital dialing was 33.1% in 2003, 20.8% in 2005, and 24.2% in 2008; while the response rate for regular mail administration was 40.0% in 2008, 36.7% in 2011, 35.2% in 2013, and 32.4% in 2017. Further information on data collection, weighted methodologies, and sample frames are available through HINTS methodology reports [11].

### Dependent Variables

Our primary objective was to examine the relationship between rural and urban residence and self-management of ePHI online among cancer patients. The original survey item of interest (survey years 2003, 2005, 2008, 2011, and 2013) is as follows:

- “In the last 12 months, have you used the internet to keep track of protected health information, such as care received, test results, or upcoming medical appointments?”

In 2017, more granular items were asked of respondents, and the survey item was changed:

- “In the past 12 months, have you used a computer, smartphone, or other electronic means to do any of the following?
  Make appointments with a health care provider
  Track health care charges and costs
  Fill out forms or paperwork related to your health care
  Look up test results”

Any respondent who answered yes to any of these subitems were categorized has having managed their ePHI online; conversely, respondents who answered no to all 4 subitems were considered to have not managed their ePHI. Before 2017, the question was asked of individuals who previously stated they had regular internet access. In 2017, the question was asked of those who stated they had both regular internet access and access to their electronic health records.

Our secondary objective was to determine whether a difference existed between rural and urban cancer patients in terms of communicating online with their health care provider. The item used in the earlier HINTS deliveries (2003-2013) is as follows:

- “In the last 12 months, have you used email or the internet to communicate with a doctor or doctor’s office?”

In 2017, the wording has changed slightly:

- “In the past 12 months, have you used a computer, smartphone, or other electronic means to do any of the following?
  Use email or the internet to communicate with a doctor or a doctor’s office.”

Before 2017, the item was only asked of those who stated they had access to the internet. In 2017, the question was asked of all participants, regardless of access to the internet or their EHRs.

### Independent Variables

Analyses were restricted to respondents who replied yes in response to the survey item “Have you ever been diagnosed as having cancer?” Additional independent variables included in analyses were age, race/ethnicity, income, gender, and educational level; all were categorical. Age was divided into age groups of 18-34, 35-49, 50-64, 65-74, and 75+. Race/ethnicity was condensed into Hispanic, non-Hispanic white, non-Hispanic black, and non-Hispanic other [12]. Income was organized into 5 categories: <$20,000, $20,000 to <$35,000, $35,000 to <$50,000, $50,000 to <$75,000, and >$75,000. Sex was categorized as a binary variable (male or female). Educational level was categorized as less than high school, high school, some college, and college graduate or higher.

Each participant was categorized as being in an urban or rural population following the Rural-Urban Continuum Code (RUCC) through the United States Department of Agriculture [13]. The code categorizes respondents based on their location (population size, metro county, or nonmetro county). The codes are on a scale of 1 to 9; if a region falls under codes 1 to 3, the classification is a metro county with a population of at least 250,000—in other words, an urban category. If a region falls under codes 4 to 9, the classification is a nonmetro county with a population ranging from 2500 to 20,000 individuals—therefore, a rural county.

### Statistical Analysis

The use of SAS 9.4 (SAS Institute Inc) allowed for weighted analysis to incorporate jackknife replicate weights to obtain population-level estimates. Briefly, a set of 50 jackknife weights are developed for each survey administration using data from the most recent US Census; this allows the weights to be used in conjunction with survey procedures within SAS to generate population-level estimates based on the survey sample data. Bivariate analyses were conducted to determine whether associations existed between geographic location and each of the independent and dependent variables; this served as an unadjusted analysis. The independent variables previously mentioned (age, race/ethnicity, income, gender, and educational level) were adjusted for using multivariable logistic regression for each dependent variable of interest. Predicted marginals were also calculated to observe any statistical differences over a period of time by adding interaction terms between each independent variable and survey year to the multivariate model one at a time. A complete case analysis was used for both outcomes of interest.

## Results

### Study Population Characteristics

All percentages reported are weighted to generate population-level estimates using the HINTS jackknife weighting paradigm. A total of 4163 respondents included across HINTS survey administrations reported having been diagnosed with cancer; this included skin cancers. These individuals had higher incomes (883/3498, or 27.6%, reported annual incomes of $75,000 or higher); were aged 50 years and older (3500/4107, 80.6%); female (2618/4121, 59.2%); and non-Hispanic white (3223/3888, 82.4%). Bivariate analyses showed a statistically significant relationship between sociodemographic characteristics (race/ethnicity, education level, income, and email/documentation) and urban/rural residency status (Table 1).

**Table 1:** Association between urban/rural status, sociodemographic characteristics, and health information technology use among cancer patients who participated in the Health Information National Trends Survey in 2003, 2005, 2008, 2011, 2013, and 2017 and reported a prior cancer diagnosis (n=4163). Row percentages are weighted to reflect United States population-level estimates.

Characteristic		Rural, n (%)	Urban, n (%)	*P* value
**Sex**				.68
	Male	287 (18.9)	126 (81.1)	
	Female	510 (19.7)	2108 (80.3)	
**Age in years**				.34
	18-34	27 (19.7)	107 (80.3)	
	35-49	92 (18.4)	381 (81.6)	
	50-64	243 (17.6)	1065 (82.4)	
	65-74	219 (22.5)	894 (77.5)	
	≥75	213 (19.4)	866 (80.6)	
**Race/ethnicity**				<.001
	Hispanic	13 (10.4)	198 (89.6)	
	Non-Hispanic white	678 (21.0)	2545 (79.0)	
	Non-Hispanic black	27 (7.6)	253 (92.4)	
	Non-Hispanic other	29 9 (16.8)	145 (83.2)	
**Education**				<.001
	Less than high school	119 (26.1)	304 (73.9)	
	High school graduate	263 (24.1)	821 (75.9)	
	Some college	189 (16.3)	970 (83.7)	
	College graduate	207 (14.2)	1192 (85.8)	
**Income**				<.001
	<$20,000	195 (26.0)	601 (74.0)	
	$20,000-<$35,000	152 (21.0)	537 (79.0)	
	$35,000-<$50,000	109 (24.0)	419 (76.0)	
	$50,000-<$75,000	117 (19.1)	485 (80.9)	
	$75,000+	99 (10.2)	784 (89.8)	
**Email/documentation**				<.001
	Yes	57 (10.4)	500 (89.6)	
	No	340 (18.9)	1589 (81.2)	
**Made appointments**				.06
	Yes	22 (13.1)	170 (86.9)	
	No	55 (22.0)	247 (78.0)	
**Tracked health costs**				.34
	Yes	16 (14.2)	116 (85.8)	
	No	59 (18.8)	299 (81.2)	
**Completed forms**				.28
	Yes	24 (15.0)	116 (85.0)	
	No	53 (20.2)	255 (79.8)	
**Test results**				.17
	Yes	24 (13.7)	160 (86.3)	
	No	52 (20.1)	262 (79.9)	
**Survey year**				.03
	2003	628 (2.1)	3354 (11.3)	
	2005	618 (2.4)	2626 (11.0)	
	2008	835 (2.3)	4243 (13.3)	
	2011	419 (2.8)	2495 (15.8)	
	2013	303 (3.1)	1981 (15.5)	
	2017	345 (3.1)	2188 (17.3)	

### Electronic Personal Health Information Use Among Rural and Urban Cancer Patients

After adjusting for sex, age, race/ethnicity, education, income, and survey year, no statistically significant association was observed between ePHI use and the urban/rural status of the cancer patients (Table 2). Urban cancer patients accessed ePHI more frequently than rural cancer patients over multiple administrations of HINTS. The only association that persisted after adjustment is between the use of ePHI among cancer patients and the survey year (*P*<.001). Although a statistically significant association was found between these two variables, the confidence intervals suggest there is no association preset between the survey year and cancer patient geography (2011: odds ratio [OR] 1.57, 95% CI 1.02-2.43; 2013: OR 3.38, 95% CI 1.89-6.15; 2017: OR 13.07, 95% CI 8.23-20.75). No association was found between ePHI use and sex, age, income, or race/ethnicity (Table 2). Additionally, there was no statistically significant association between ePHI use and education (*P*=.07).

**Table 2:** Logistic regression model of electronic personal health information use among patients who reported being diagnosed with cancer grouped by rural and urban status based from the Health Information National Trends Survey (n=1388) in the years 2008, 2011, 2013, and 2017, adjusted for sex, age, race/ethnicity, education, and income.

Characteristics		Odds ratio (95% CI)	*P* value
**Residential area**			.40
	Urban	Ref^a^	
	Rural	0.78 (0.43-1.40)	
**Sex**			.75
	Female	Ref	
	Male	0.93 (0.60-1.45)	
**Age in years**			.52
	18-34	Ref	
	35-49	0.50 (0.17-1.47)	
	50-64	0.48 (0.19-1.22)	
	65-74	0.42 (0.16-1.11)	
	≥75	0.41 (0.13-1.27)	
**Race/ethnicity**			.27
	Non-Hispanic white	Ref	
	Hispanic	0.94 (0.46-1.93)	
	Non-Hispanic black	1.16 (0.51-2.64)	
	Non-Hispanic other	2.05 (0.98-4.31)	
**Education**			.07
	Less than high school	Ref	
	High school graduate	1.03 (0.19-5.50)	
	Some college	2.01 (0.38-10.70)	
	College graduate	2.04 (0.41-10.03)	
**Income**			.09
	<$20,000	Ref	
	$20,000-<$35,000	1.50 (0.62-3.60)	
	$35,000-<$50,000	1.13 (0.48-2.69)	
	$50,000-<$75,000	1.93 (0.72-5.15)	
	$75,000+	2.21 (0.90-5.40)	
**Survey year**			<.001
	2008	Ref	
	2011	1.57 (1.02-2.43)	
	2013	3.38 (1.89-6.15)	
	2017	13.07 (8.23-20.75)	

^a^Ref: reference.

### Email Contact With Providers Among Rural and Urban Patients

Rural cancer patients had a 0.52-fold decreased odds of emailing their health care providers as compared with urban cancer patients, adjusting for gender, age, race/ethnicity, education, income, and survey year (95% CI 0.32-0.84, *P*=.009, Table 3). There were statistically significant associations between email communication with providers and age (*P*=.03), survey year (*P*<.001), and education (*P*=.002); however, confidence intervals for educational levels indicated no statistically significant difference. The association with email communication between cancer patients and health care providers increased with each survey administration. As the age of the respondents increased, respondents were less likely to have communication with their health care provider (Table 3).

**Table 3:** Logistic regression model of email communication between patients who reported being diagnosed with cancer and health care provider grouped by rural/urban status based on responses from the Health Information National Trends Survey (n=2058) in the years 2003, 2005, 2008, 2011, 2013, and 2017. Adjusted for gender, age, race/ethnicity, education, and income.

Characteristic		Odds ratio (95% CI)	*P* value
**Residential area**			.009
	Urban	Ref^a^	
	Rural	0.52 (0.32-0.84)	
**Sex**			.22
	Female	Ref	
	Male	1.20 (0.89-1.61)	
**Age in years**			.03
	18-34	Ref	
	35-49	0.33 (0.14-0.77)	
	50-64	0.39 (0.17-0.88)	
	65-74	0.26 (0.11-0.61)	
	≥75	0.25 (0.10-0.64)	
**Race/ethnicity**			.97
	Non-Hispanic white	Ref	
	Hispanic	0.86 (0.33-2.24)	
	Non-Hispanic black	0.91 (0.42-2.01)	
	Non-Hispanic other	1.13 (0.62-2.05)	
**Education**			.002
	Less than high school	Ref	
	High school graduate	1.06 (0.35-3.18)	
	Some college	2.61 (0.90-7.51)	
	College graduate	2.60 (0.87-7.77)	
**Income**			.10
	<$20,000	Ref	
	$20,000-<$35,000	0.88 (0.45-1.72)	
	$35,000-<$50,000	0.95 (0.49-1.82)	
	$50,000-<$75,000	1.23 (0.64-2.37)	
	$75,000+	2.01 (0.97-4.14)	
**Survey year**			<.001
	2003	Ref	
	2005	1.57 (0.96-2.57)	
	2008	1.95 (1.12-3.40)	
	2011	3.02 (1.68-5.44)	
	2013	7.78 (4.51-13.41)	
	2017	8.45 (5.15-13.83)	

^a^Ref: reference.

## Discussion

### Principal Findings

In this study, we sought to determine whether an association existed between ePHI use and rural/urban residence status among cancer patients. Additionally, we sought to examine whether a relationship exists between email communication with health care providers and rural/urban residence among cancer patients. No association was found between ePHI use and geography among cancer patients; this lack of association persisted after adjustment for relevant sociodemographic covariates. However, results did show that rural cancer patients were significantly less likely to email health care providers compared with their urban counterparts.

Prior work has shown no difference in the use of ePHI tools between urban and rural residents in the general population [14]. Our adjusted results lead us to conclude that there is no difference in use of ePHI tools between rural and urban cancer patients, indicating our original hypothesis that a difference existed was incorrect. Rural cancer patients may access their ePHI less than urban cancer patients. There may be several underlying reasons for this trend. First, lower rates of ePHI use may be due in part to lack of awareness; for example, health care providers tend to offer rural patients access to ePHI tools less frequently, which may play a role further exacerbating the digital divide among rural and urban patients [14]. Second, despite advances in the internet and technology, patients in rural regions are at a disadvantage in comparison with patients in urban regions. Individuals who live in a rural region are reported to have lower use of the internet than individuals who live in an urban region [8]. Due to the lack of infrastructure of telecommunication, rural regions typically do not have optimal internet service [15]. This trend persists despite the Federal Communications Commission effort to expand broadband access [15]. A lack of internet access may prevent patients in rural regions from accessing ePHI tools that could potentially improve their quality of care, further perpetuating the existing health information technology divide.

While no association was found between rural and urban residency and ePHI use, a statistically significant association was found between geography and emailing providers. A recent study using HINTS data found a similar disparity among all HINTS participants, with rural participants reporting that they emailed their health care providers significantly less than urban counterparts [12]. While some have hypothesized that individuals with chronic conditions, such as cancer, are more likely to email their health care providers, patients with one or more chronic conditions have actually been shown to have reduced odds of emailing their providers [16]. The results presented here suggest that this disparity may be even more exacerbated among rural patients with chronic conditions. Additional studies are needed to further characterize the barriers to use of email to communicate with providers; we hypothesize that these may include personal factors (such as lack of awareness, unwillingness to adopt ePHI-related technologies, and/or concerns about privacy) as well as structural factors (lack of access to reliable internet connections, cellular networks, etc).

### Strengths and Limitations

A strength of this study is its use of HINTS. This is a nationally representative survey of individuals who are 18 years or older that has been administered several times over the course of 15 years, allowing for longitudinal study of trends. An additional strength is its use of a jackknife weighting paradigm that allows for the generation of population-level estimates. A limitation to this study is that the items analyzed addressing ePHI and email communication were fairly general; this may have limited the ability to identify specific relationships, included the expected ones. Another limitation to this study is smaller sample size in some categories, due to the restriction of the data solely to cancer patients, as well as the inability to determine causation due to the cross-sectional nature of each survey. Furthermore, HINTS did not ask follow-up questions about the frequency of ePHI use and communication in older survey administrations, nor did the survey include items regarding which provider was emailed and what type of online tool was used to carry out these tasks.

### Conclusions and Future Directions

We sought to assess the use of ePHI tools and frequency of electronic communication between adult cancer patients and their health care providers and to determine whether a difference existed in use between those living in rural and urban areas of the United States. Although our results demonstrate that there is no statistically significant difference between the rural/urban status of cancer patients and their ePHI use, the data lead us to believe that rural cancer patients access their electronic records less frequently than urban cancer patients. Cancer patients in urban regions are also more likely to communicate with their health care providers via email than rural cancer patients. Although our results demonstrate a relationship present for both email communication and ePHI use, there are many other components that affect the role of internet access and use of these tools that we could not explore due to the limitations present. By increasing the awareness, access, and use of these tools in rural populations, there is the potential to improve the patients’ ability to increase self-efficacy with regard to their health care and improve clinical outcomes. Future studies should focus on targeted interventions for rural cancer patients and examine whether the implementation of ePHI and electronic messaging tools affects patient outcomes.

## Abbreviations

EHR: electronic health record
ePHI: electronic personal health information
HINTS: Health Information National Trends Survey
OR: odds ratio
RDD: random digital dialing
RUCC: Rural-Urban Continuum Code
